# Crystal structure and DFT study of 2-(pyren-1-yl)-1*H*-benzimidazole

**DOI:** 10.1107/S2056989017010271

**Published:** 2017-07-17

**Authors:** Md. Serajul Haque Faizi, Necmi Dege, S. Malinkin

**Affiliations:** a Department of Chemistry, College of Science, Sultan Qaboos University, PO Box 36 Al-Khod 123, Muscat, Sultanate of Oman; b Ondokuz Mayıs University, Arts and Sciences Faculty, Department of Physics, 55139 Atakum–Samsun, Turkey; c Department of Chemistry, National Taras Shevchenko University of Kiev, 64/13, Volodymyrska Street, City of Kyiv 01601, Ukraine

**Keywords:** crystal structure, pyrene-1-carbaldehyde, *o*-phenyl­enedi­amine, benzimidazole, N—H⋯N hydrogen bonding, C—H⋯π inter­actions, DFT

## Abstract

In the title compound, C_23_H_14_N_2_, (**I**), the dihedral angle between the mean planes of the pyrene and benzimidazole ring systems is 42.08 (5)°, with a bridging C—C bond length of 1.463 (3) Å. In the crystal, mol­ecules are linked by N—H⋯N hydrogen bonds, forming columns propagating along the *b*-axis direction. The columns are linked *via* C—H⋯π inter­actions, forming slabs parallel to the *ab* plane. There are no significant π–π inter­actions present in the crystal structure. The density functional theory (DFT) optimized structure, at the B3LYP/ 6-311G(d,p) level, is compared with the experimentally determined solid-state structure of the title compound.

## Abstract 2

The title compound was prepared from an equimolar mixuture of *o*-phenyl­enedi­amine and pyrene-1-carboxaldehyde. We report herein on its crystal structure and a density functional theory (DFT) study.

## Chemical context   

Benzimidazoles, which are analogues of imidazole contained in histidine, are an important class of biologically active compounds (Collman *et al.*, 1973 ▸). In addition, they are excellent organic ligands of many metal ions (Sundberg & Martin, 1974 ▸). The pyrene unit is one of the most commonly used fluoro­phores due to its strong luminescence and chemical stability (Aoki *et al.*, 1991 ▸; Nishizawa *et al.*, 1999 ▸; van der Veen *et al.*, 2000 ▸). Another inter­esting feature of the pyrene unit is the inter­action between the pyrene aromatic rings in the crystal packing, which can permit the formation of highly ordered mol­ecular aggregates in the solid state by architecturally controlled self-assembly (Desiraju & Gavezzotti, 1989 ▸; Munakata *et al.*, 1994 ▸). Pyrene is a commonly used fluoro­phore due to its unusual fluorescence properties, *viz*. intense fluorescence signals and vibronic band dependence with the media (Karpovich & Blanchard, 1995 ▸), and has been used in fluorescence sensors (Bell & Hext, 2004 ▸) and excimer formation (Lodeiro *et al.*, 2006 ▸). As a result of these particular properties and because of its chemical stability, it is also employed as a probe for solid-state studies and polymer association (Seixas de Melo *et al.*, 2003 ▸).
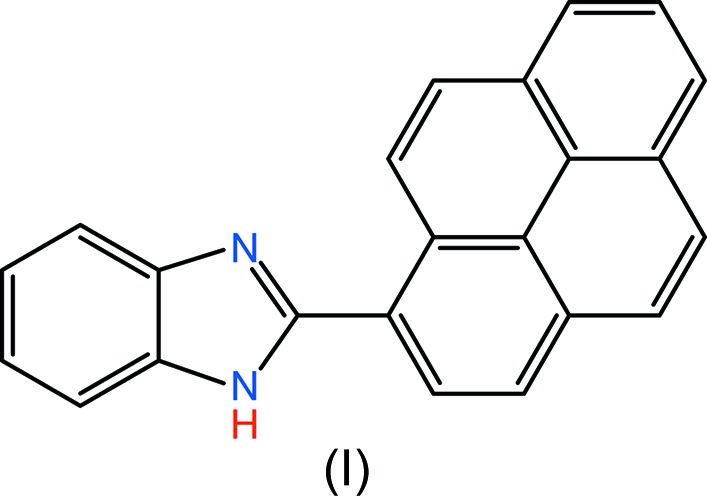


The title compound was prepared from an equimolar mixuture of 1:1 *o*-phenyl­enedi­amine and pyrene-1-carbaldehyde. Synthesis and characterization of many benzimidazole-ring-containing compounds have been reported (Yan *et al.*, 2009 ▸; Hallett *et al.*, 2012 ▸; Xia *et al.*, 2014 ▸; Dhanalakshmi *et al.*, 2014 ▸; Guo *et al.*, 2015 ▸; Song *et al.*, 2010 ▸), but very few compounds have been structurally characterized. Previously, Zhao *et al.* (2016 ▸) reported on the synthesis of 2-(pyren-1-yl)benzimidazole, used as a fluorescent probe for the detection of iron(III) ions in aqueous solution, but gave no structural details of the compound. The present work is part of an ongoing structural study of pyrene-ring-system derivatives (Faizi & Prisyazhnaya, 2015 ▸). The results of the calculations by density functional theory (DFT) on (**I**), carried out at the B3LYP/6-311G(d,p) level, are compared with the experimentally determined mol­ecular structure in the solid state.

## Structural commentary   

**Figure 1:**
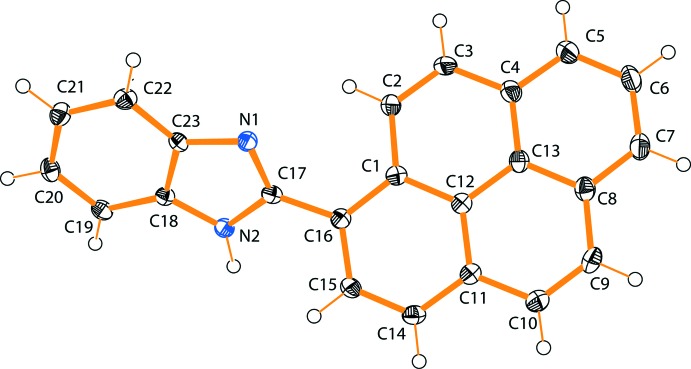
The mol­ecular structure of compound (**I**), with the atom labelling. Displacement ellipsoids are drawn at the 40% probability level.

The mol­ecular structure of the title compound, (**I**), is illustrated in Fig. 1 ▸. The compound is nonplanar, the rotation around the bond connecting the two aromatic moieties, which is predominantly σ in character [C16—C17 = 1.463 (3) Å], being described by the torsion angle N1—C17—C16—C1 of −39.49 (10)°. The mean planes of the pyrene (atoms C1–C16; r.m.s. deviation = 0.038 Å) and benzimidazole (N1/N2/C17–C23) ring systems are inclined to one another by 42.08 (5)°, reflecting the significant deviation from overall mol­ecular planarity.

## Supra­molecular features   

**Figure 2:**
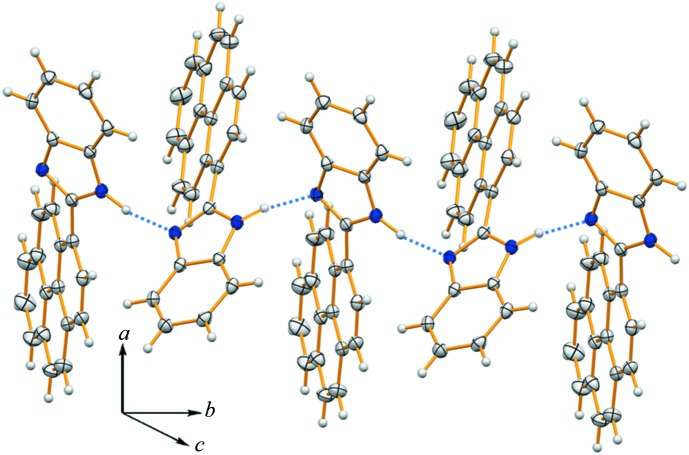
A view of the N—H⋯N hydrogen-bonded column (dashed lines; Table 1 ▸) in the crystal of compound (**I**), propagating along the *b*-axis direction.

**Figure 3:**
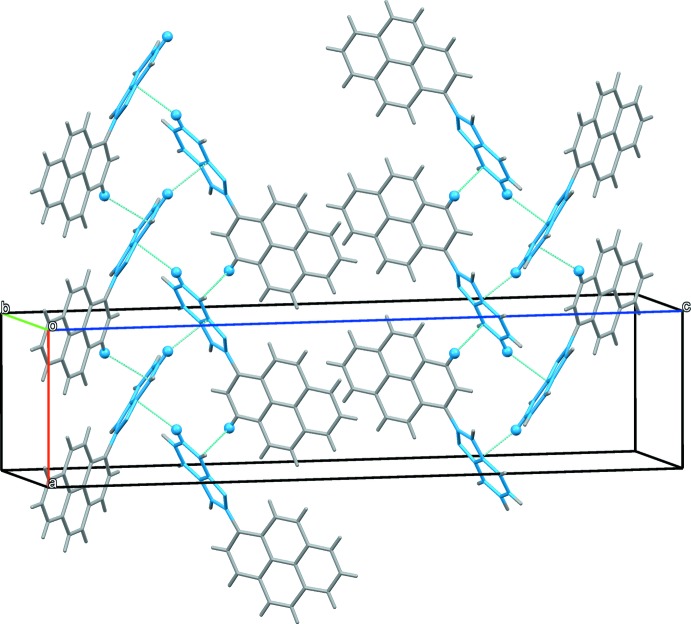
A view along the *b* axis of the crystal packing of compound (**I**). The C—H⋯π inter­actions are illustrated by dashed lines (Table 1 ▸).

In the crystal of (**I**), mol­ecules are assembled *via* N2—H⋯N1^i^ hydrogen bonds (Table 1 ▸) into columns propagating along the *b*-axis direction (Fig. 2 ▸). The columns are linked by C—H⋯π inter­actions (Table 1 ▸), forming slabs parallel to the *ab* plane (Fig. 3 ▸).

## Database survey   

A search of the Cambridge Structural Database (CSD, Version 5.38, last update May 2017; Groom *et al.*, 2016 ▸) gave a number of hits for similar compounds, *viz.* phenyl-2-benzimidazole derivatives (**II**) (CSD refcode FOBZUS; Li *et al.*, 2005 ▸) and (**III**) (LIYTIW; Bei *et al.*, 2000 ▸), and phenanthro­imidazole derivatives (**IV**) (ERODOE; Bu *et al.*, 2003 ▸) and (**V**) (SUZHIE; Krebs *et al.*, 2001 ▸). All four organic compounds are nonplanar and have a similar C—C bond length between the aromatic ring systems. In (**I**), this bond (C16—C17) is 1.463 (3) Å, and the two ring systems are inclined to one another by 42.08 (5)°. These values are close to those reported for compounds (**II**) (1.474 Å and 40.17°), (**III**) (1.467 Å and 31.12°) and (**V**) (1.436 and 30.12°), but the anthracene–phenanthro­imidazole compound (**IV**) has a larger deviation from planarity, with the two aromatic ring systems being almost perpendicular to one another (1.488 Å and 76.54°) due to significant steric hindrance of the anthracene moiety. Two other compounds are worth mentioning, *viz.* 9-(1*H*-benzimidazol-2-yl)-2,3,6,7-tetra­hydro-1*H*,5*H*-pyrido[3,2,1-*ij*]quinoline (**VI**) (TAQHUR; Gonzalez & Unnamatla, 2017 ▸) and 2-(pyren-1-yl)-1*H*-phenanthro[9,10-*d*]imidazole unknown solvate (**VII**) (KUFLOO; Subeesh *et al.*, 2015 ▸). In (**VI**), the mean plane of the pyrido­quinoline moiety is inclined to the benzimidole ring system by 37.94 (10)° and the bridging C—C bond is 1.467 (3) Å, similar to the situation in (**I**). In (**VII**), the mean plane of the pyrene ring system is inclined to the phenanthro­imidazole mean plane by 63.37 (6)° and the bridging C—C bond is 1.463 (5) Å. As in (**IV**), this large dihedral angle is due to steric hinderance.

## DFT study   

The DFT quantum-chemical calculations were performed at the B3LYP/6-311G(d,p) level (Becke, 1993 ▸), as implemented in *GAUSSIAN09* (Frisch *et al.*, 2009 ▸). DFT structure optimization of (**I**) was performed starting from the X-ray geometry and the values compared with experimental values (see Table 2 ▸). From these results we can conclude that basis set 6-311G(d,p) is well suited in its approach to the experimental data.

**Figure 4:**
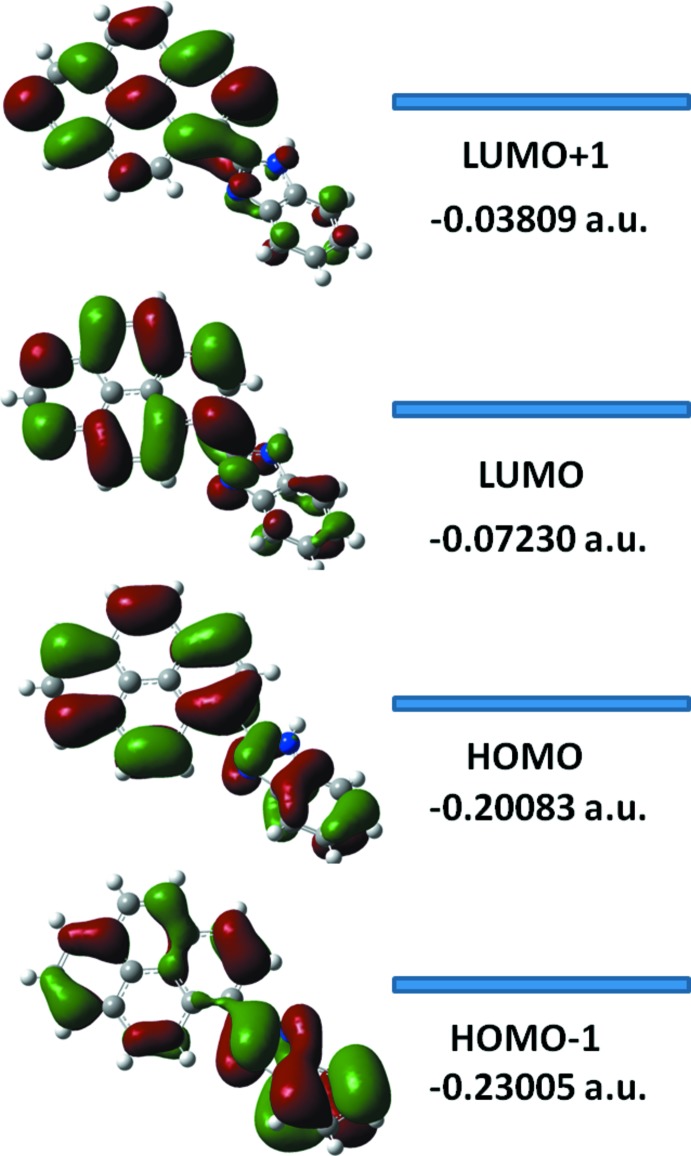
Electron distribution of the HOMO-1, HOMO, LUMO and LUMO+1 energy levels for compound (**I**).

The DFT study of (**I**) shows that the HOMO and LUMO are localized in the plane extending from the whole pyrene ring to the benzimidazole ring. The electron distribution of the HOMO-1, HOMO, LUMO and the LUMO+1 energy levels are shown in Fig. 4 ▸. The mol­ecular orbital of HOMO contains both σ and π character, whereas HOMO-1 is dominated by orbital density. The LUMO is mainly composed of density, while LUMO+1 has both σ and π character and electronic density. The HOMO–LUMO gap was found to be 0.273 a.u. and the frontier mol­ecular orbital energies, *E*_HOMO_ and *E*_LUMO_, were −0.20083 and −0.07230 a.u., respectively.

## Synthesis and crystallization   

Pyrene-1-carbaldehyde (0.2306 g, 1.0 mmol) was added to a 50 ml round-bottomed flask containing 10 ml of CH_2_Cl_2_. Then a 10 ml CH_2_Cl_2_ solution containing 0.1080 g (1.0 mmol) *o*-phenyl­enedi­amine was added dropwise over a period of 30 min with stirring. The mixture was stirred at room temperature for 48 h. The solvent was then evaporated and the residue purified by aluminium oxide gel-column chromatography using CH_2_Cl_2_ as the eluent to obtain a pale-yellow powder of (**I**) (yield 0.2311 g, 72.6%). Colourless prismatic crystals were obtained by slow evaporation of a solution of (**I**) from methanol.

## Refinement   

Crystal data, data collection and structure refinement details are summarized in Table 3 ▸. The N-bound H atoms were located in a difference Fourier map and refined with *U*_iso_(H) = 1.2*U*_eq_(N). The C-bound H atoms were included in calculated positions and refined as riding, with C—H = 0.93–0.96 Å and *U*_iso_(H) = 1.2*U*_eq_(C).

## Supplementary Material

Crystal structure: contains datablock(s) I, Global. DOI: 10.1107/S2056989017010271/su5378sup1.cif

Structure factors: contains datablock(s) I. DOI: 10.1107/S2056989017010271/su5378Isup2.hkl

Supporting information file. DOI: 10.1107/S2056989017010271/su5378Isup3.cml

CCDC reference: 1547858

Additional supporting information:  crystallographic information; 3D view; checkCIF report

## Figures and Tables

**Table 1:** Hydrogen-bond geometry (Å, °) *Cg*1, *Cg*6 and *Cg*7 are the centroids of rings N1/N2/C17/C18/C23, C18-C23 and N1/N2/C17-C23.

*D*—H⋯*A*	*D*—H	H⋯*A*	*D*⋯*A*	*D*—H⋯*A*
N2—H1*A*⋯N1^i^	0.94 (2)	1.92 (2)	2.838 (2)	164 (2)
C14—H14⋯*Cg*6^ii^	0.93	2.83	3.537 (2)	134
C21—H21⋯*Cg*1^iii^	0.93	2.95	3.605 (2)	129
C21—H21⋯*Cg*7^iii^	0.93	2.84	3.618 (2)	142

Symmetry codes: (i) 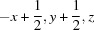; (ii) 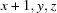; (iii) 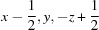.

**Table 2:** Comparison of selected geometric data for (**I**) (Å, °) from X-ray and calculated (DFT) data

Bonds/angles	X-ray	B3LYP/6-311G(d,p)
C17—N2	1.364 (2)	1.365
C18—N2	1.376 (2)	1.375
C17—N1	1.330 (2)	1.329
C23—N1	1.389 (2)	1.389
C17—C16	1.463 (3)	1.462
C16—C17—N2	121.57 (17)	121.51
C16—C17—N1	125.73 (17)	125.82
N1—C17—N2	112.48 (17)	112.44

**Table 3:** Experimental details

Crystal data	
Chemical formula	C_23_H_14_N_2_
*M*_r_	318.36
Crystal system, space group	Orthorhombic, *P* *b* *c* *a*
Temperature (K)	273
*a*, *b*, *c* (Å)	8.7344 (8), 9.5967 (9), 36.410 (3)
*V* (Å^3^)	3052.0 (5)
*Z*	8
Radiation type	Mo *K*α
μ (mm^−1^)	0.08
Crystal size (mm)	0.65 × 0.43 × 0.32

Data collection	
Diffractometer	Bruker APEXII CCD area detector
No. of measured, independent and observed [*I* > 2σ(*I*)] reflections	36046, 2986, 1951
*R*_int_	0.103
(sin θ/λ)_max_ (Å^−1^)	0.617

Refinement	
*R*[*F* ^2^ > 2σ(*F* ^2^)], *wR*(*F* ^2^), *S*	0.049, 0.113, 1.03
No. of reflections	2986
No. of parameters	230
H-atom treatment	H atoms treated by a mixture of independent and constrained refinement
Δρ_max_, Δρ_min_ (e Å^−3^)	0.19, −0.26

Computer programs: *APEX2* (Bruker, 2005 ▸), *SAINT* (Bruker, 2005 ▸), *SHELXT2014* (Sheldrick, 2015*a* ▸), *SHELXTL* (Sheldrick, 2008 ▸), *SHELXL2016* (Sheldrick, 2015*b* ▸) and *PLATON* (Spek, 2009 ▸).

## supplementary crystallographic information

### Crystal data

**Table:**

C_23_H_14_N_2_	*D*_x_ = 1.386 Mg m^−^^3^
*M**_r_* = 318.36	Mo *K*α radiation, λ = 0.71073 Å
Orthorhombic, *P**b**c**a*	Cell parameters from 1528 reflections
*a* = 8.7344 (8) Å	θ = 2.4–16.1°
*b* = 9.5967 (9) Å	µ = 0.08 mm^−^^1^
*c* = 36.410 (3) Å	*T* = 273 K
*V* = 3052.0 (5) Å^3^	Prism, colorless
*Z* = 8	0.65 × 0.43 × 0.32 mm
*F*(000) = 1328	

### Data collection

**Table:**

Bruker APEXII CCD area detector diffractometer	1951 reflections with *I* > 2σ(*I*)
Radiation source: sealed tube	*R*_int_ = 0.103
Graphite monochromator	θ_max_ = 26.0°, θ_min_ = 2.6°
phi and ω scans	*h* = −10→10
36046 measured reflections	*k* = −11→11
2986 independent reflections	*l* = −44→44

### Refinement

**Table:**

Refinement on *F*^2^	Primary atom site location: structure-invariant direct methods
Least-squares matrix: full	Secondary atom site location: difference Fourier map
*R*[*F*^2^ > 2σ(*F*^2^)] = 0.049	Hydrogen site location: inferred from neighbouring sites
*wR*(*F*^2^) = 0.113	H atoms treated by a mixture of independent and constrained refinement
*S* = 1.03	*w* = 1/[σ^2^(*F*_o_^2^) + (0.0467*P*)^2^ + 1.2809*P*] where *P* = (*F*_o_^2^ + 2*F*_c_^2^)/3
2986 reflections	(Δ/σ)_max_ < 0.001
230 parameters	Δρ_max_ = 0.19 e Å^−^^3^
0 restraints	Δρ_min_ = −0.26 e Å^−^^3^

### Special details

**Table:**

Geometry. All esds (except the esd in the dihedral angle between two l.s. planes) are estimated using the full covariance matrix. The cell esds are taken into account individually in the estimation of esds in distances, angles and torsion angles; correlations between esds in cell parameters are only used when they are defined by crystal symmetry. An approximate (isotropic) treatment of cell esds is used for estimating esds involving l.s. planes.

### Fractional atomic coordinates and isotropic or equivalent isotropic displacement parameters (Å^2^)

**Table:**

	*x*	*y*	*z*	*U*_iso_*/*U*_eq_	
N1	0.15929 (18)	0.66443 (16)	0.32741 (4)	0.0190 (4)	
N2	0.21591 (19)	0.89171 (17)	0.32393 (5)	0.0195 (4)	
C23	0.0470 (2)	0.73209 (19)	0.30713 (5)	0.0176 (5)	
C17	0.2581 (2)	0.7637 (2)	0.33673 (5)	0.0190 (5)	
C16	0.4042 (2)	0.74213 (19)	0.35556 (5)	0.0195 (5)	
C18	0.0808 (2)	0.8748 (2)	0.30499 (5)	0.0179 (4)	
C1	0.4229 (2)	0.6448 (2)	0.38432 (5)	0.0193 (5)	
C12	0.5724 (2)	0.6212 (2)	0.39850 (5)	0.0199 (5)	
C11	0.6999 (2)	0.6952 (2)	0.38411 (5)	0.0210 (5)	
C19	−0.0099 (2)	0.9680 (2)	0.28557 (5)	0.0214 (5)	
H19	0.012157	1.062811	0.284937	0.026*	
C20	−0.1341 (2)	0.9132 (2)	0.26728 (5)	0.0235 (5)	
H20	−0.195476	0.971887	0.253275	0.028*	
C13	0.5956 (2)	0.5241 (2)	0.42759 (5)	0.0220 (5)	
C2	0.2978 (2)	0.5706 (2)	0.40072 (6)	0.0235 (5)	
H2	0.198943	0.585875	0.392152	0.028*	
C8	0.7452 (2)	0.4990 (2)	0.44151 (5)	0.0252 (5)	
C14	0.6749 (2)	0.7950 (2)	0.35696 (6)	0.0239 (5)	
H14	0.756769	0.847263	0.348192	0.029*	
C4	0.4691 (2)	0.4519 (2)	0.44303 (6)	0.0253 (5)	
C15	0.5309 (2)	0.8170 (2)	0.34299 (6)	0.0231 (5)	
H15	0.517365	0.883532	0.324701	0.028*	
C22	−0.0814 (2)	0.6796 (2)	0.28931 (5)	0.0224 (5)	
H22	−0.106657	0.585627	0.290863	0.027*	
C21	−0.1703 (2)	0.7709 (2)	0.26926 (5)	0.0247 (5)	
H21	−0.255742	0.737591	0.256826	0.030*	
C10	0.8497 (2)	0.6653 (2)	0.39798 (6)	0.0273 (5)	
H10	0.933899	0.711258	0.388120	0.033*	
C9	0.8707 (2)	0.5716 (2)	0.42512 (6)	0.0292 (5)	
H9	0.969483	0.553670	0.433385	0.035*	
C3	0.3207 (2)	0.4788 (2)	0.42841 (6)	0.0271 (5)	
H3	0.236939	0.431824	0.438185	0.033*	
C7	0.7635 (3)	0.4060 (2)	0.47055 (6)	0.0311 (6)	
H7	0.860961	0.389193	0.479795	0.037*	
C5	0.4941 (3)	0.3609 (2)	0.47218 (6)	0.0318 (6)	
H5	0.411550	0.314383	0.482643	0.038*	
C6	0.6397 (3)	0.3386 (2)	0.48582 (6)	0.0357 (6)	
H6	0.654109	0.277746	0.505401	0.043*	
H1A	0.271 (2)	0.975 (2)	0.3280 (6)	0.033 (6)*	

### Atomic displacement parameters (Å^2^)

**Table:**

	*U*^11^	*U*^22^	*U*^33^	*U*^12^	*U*^13^	*U*^23^
N1	0.0191 (9)	0.0153 (9)	0.0225 (9)	0.0003 (7)	−0.0016 (8)	0.0003 (8)
N2	0.0203 (10)	0.0129 (9)	0.0253 (9)	−0.0010 (8)	−0.0014 (8)	0.0010 (7)
C23	0.0207 (11)	0.0152 (10)	0.0170 (10)	0.0012 (8)	0.0014 (9)	0.0013 (8)
C17	0.0198 (11)	0.0168 (11)	0.0205 (10)	0.0007 (9)	0.0039 (10)	0.0006 (8)
C16	0.0200 (11)	0.0158 (10)	0.0227 (11)	0.0009 (9)	0.0000 (10)	−0.0016 (8)
C18	0.0177 (11)	0.0180 (10)	0.0181 (10)	0.0013 (8)	0.0016 (9)	−0.0006 (8)
C1	0.0201 (11)	0.0167 (11)	0.0210 (11)	0.0009 (9)	0.0014 (9)	−0.0029 (9)
C12	0.0208 (11)	0.0187 (11)	0.0202 (10)	0.0009 (9)	0.0008 (9)	−0.0029 (9)
C11	0.0201 (11)	0.0205 (11)	0.0224 (11)	0.0009 (9)	0.0002 (9)	−0.0035 (9)
C19	0.0258 (12)	0.0151 (10)	0.0234 (11)	0.0019 (9)	0.0021 (10)	0.0015 (9)
C20	0.0254 (12)	0.0232 (12)	0.0221 (11)	0.0076 (9)	−0.0011 (10)	0.0011 (9)
C13	0.0228 (12)	0.0220 (11)	0.0213 (11)	0.0038 (9)	−0.0002 (10)	−0.0012 (9)
C2	0.0200 (12)	0.0235 (11)	0.0270 (12)	0.0024 (9)	−0.0002 (10)	0.0027 (10)
C8	0.0246 (12)	0.0265 (12)	0.0246 (11)	0.0052 (9)	−0.0009 (10)	0.0005 (9)
C14	0.0212 (12)	0.0218 (11)	0.0288 (12)	−0.0035 (9)	0.0036 (10)	0.0010 (9)
C4	0.0260 (12)	0.0237 (12)	0.0263 (12)	0.0027 (10)	0.0006 (10)	0.0027 (9)
C15	0.0260 (12)	0.0174 (11)	0.0259 (11)	0.0005 (9)	−0.0005 (10)	0.0036 (9)
C22	0.0253 (12)	0.0154 (11)	0.0267 (11)	−0.0014 (9)	−0.0005 (10)	−0.0022 (9)
C21	0.0240 (12)	0.0246 (12)	0.0256 (12)	0.0009 (9)	−0.0043 (10)	−0.0032 (9)
C10	0.0208 (12)	0.0316 (13)	0.0296 (12)	−0.0013 (10)	0.0027 (10)	0.0018 (10)
C9	0.0183 (12)	0.0361 (14)	0.0333 (13)	0.0056 (10)	−0.0041 (10)	−0.0008 (11)
C3	0.0209 (12)	0.0287 (12)	0.0317 (12)	−0.0023 (10)	0.0046 (10)	0.0075 (10)
C7	0.0259 (13)	0.0351 (13)	0.0325 (12)	0.0066 (10)	−0.0054 (11)	0.0050 (10)
C5	0.0289 (13)	0.0341 (14)	0.0326 (13)	0.0015 (11)	0.0033 (11)	0.0102 (10)
C6	0.0367 (14)	0.0382 (14)	0.0322 (13)	0.0097 (11)	−0.0022 (12)	0.0143 (11)

### Geometric parameters (Å, º)

**Table:**

N1—C17	1.330 (2)	C13—C8	1.423 (3)
N1—C23	1.389 (2)	C2—C3	1.354 (3)
N2—C17	1.364 (2)	C2—H2	0.9300
N2—C18	1.376 (2)	C8—C7	1.392 (3)
N2—H1A	0.94 (2)	C8—C9	1.429 (3)
C23—C22	1.390 (3)	C14—C15	1.373 (3)
C23—C18	1.403 (3)	C14—H14	0.9300
C17—C16	1.463 (3)	C4—C5	1.391 (3)
C16—C15	1.397 (3)	C4—C3	1.425 (3)
C16—C1	1.412 (3)	C15—H15	0.9300
C18—C19	1.389 (3)	C22—C21	1.380 (3)
C1—C12	1.422 (3)	C22—H22	0.9300
C1—C2	1.434 (3)	C21—H21	0.9300
C12—C11	1.421 (3)	C10—C9	1.349 (3)
C12—C13	1.426 (3)	C10—H10	0.9300
C11—C14	1.393 (3)	C9—H9	0.9300
C11—C10	1.431 (3)	C3—H3	0.9300
C19—C20	1.377 (3)	C7—C6	1.378 (3)
C19—H19	0.9300	C7—H7	0.9300
C20—C21	1.403 (3)	C5—C6	1.381 (3)
C20—H20	0.9300	C5—H5	0.9300
C13—C4	1.420 (3)	C6—H6	0.9300
			
C17—N1—C23	105.01 (16)	C7—C8—C13	118.9 (2)
C17—N2—C18	107.25 (16)	C7—C8—C9	122.8 (2)
C17—N2—H1A	124.7 (13)	C13—C8—C9	118.26 (18)
C18—N2—H1A	128.1 (13)	C15—C14—C11	120.81 (19)
N1—C23—C22	130.42 (18)	C15—C14—H14	119.6
N1—C23—C18	109.72 (17)	C11—C14—H14	119.6
C22—C23—C18	119.84 (18)	C5—C4—C13	119.1 (2)
N1—C17—N2	112.48 (17)	C5—C4—C3	122.8 (2)
N1—C17—C16	125.73 (17)	C13—C4—C3	118.12 (18)
N2—C17—C16	121.57 (17)	C14—C15—C16	121.67 (19)
C15—C16—C1	119.46 (18)	C14—C15—H15	119.2
C15—C16—C17	117.66 (17)	C16—C15—H15	119.2
C1—C16—C17	122.80 (18)	C21—C22—C23	118.06 (19)
N2—C18—C19	131.90 (18)	C21—C22—H22	121.0
N2—C18—C23	105.54 (16)	C23—C22—H22	121.0
C19—C18—C23	122.51 (18)	C22—C21—C20	121.24 (19)
C16—C1—C12	118.71 (18)	C22—C21—H21	119.4
C16—C1—C2	123.31 (19)	C20—C21—H21	119.4
C12—C1—C2	117.96 (18)	C9—C10—C11	121.1 (2)
C11—C12—C1	120.40 (18)	C9—C10—H10	119.4
C11—C12—C13	119.29 (18)	C11—C10—H10	119.4
C1—C12—C13	120.30 (18)	C10—C9—C8	121.8 (2)
C14—C11—C12	118.83 (18)	C10—C9—H9	119.1
C14—C11—C10	122.10 (19)	C8—C9—H9	119.1
C12—C11—C10	119.07 (18)	C2—C3—C4	122.0 (2)
C20—C19—C18	116.68 (19)	C2—C3—H3	119.0
C20—C19—H19	121.7	C4—C3—H3	119.0
C18—C19—H19	121.7	C6—C7—C8	121.2 (2)
C19—C20—C21	121.62 (19)	C6—C7—H7	119.4
C19—C20—H20	119.2	C8—C7—H7	119.4
C21—C20—H20	119.2	C6—C5—C4	121.1 (2)
C4—C13—C8	119.43 (18)	C6—C5—H5	119.5
C4—C13—C12	120.17 (18)	C4—C5—H5	119.5
C8—C13—C12	120.40 (19)	C7—C6—C5	120.3 (2)
C3—C2—C1	121.41 (19)	C7—C6—H6	119.9
C3—C2—H2	119.3	C5—C6—H6	119.9
C1—C2—H2	119.3		

### Hydrogen-bond geometry (Å, º)

Cg1, Cg6 and Cg7 are the centroids of rings N1/N2/C17/C18/C23, C18-C23 and N1/N2/C17-C23.

**Table:**

*D*—H···*A*	*D*—H	H···*A*	*D*···*A*	*D*—H···*A*
N2—H1*A*···N1^i^	0.94 (2)	1.92 (2)	2.838 (2)	164 (2)
C14—H14···*Cg*6^ii^	0.93	2.83	3.537 (2)	134
C21—H21···*Cg*1^iii^	0.93	2.95	3.605 (2)	129
C21—H21···*Cg*7^iii^	0.93	2.84	3.618 (2)	142

Symmetry codes: (i) −*x*+1/2, *y*+1/2, *z*; (ii) *x*+1, *y*, *z*; (iii) *x*−1/2, *y*, −*z*+1/2.
